# Music Therapy and Korsakoff’s Syndrome: The State of the Art

**DOI:** 10.3390/jcm12144609

**Published:** 2023-07-11

**Authors:** Monique van Bruggen-Rufi, Gerjanne van der Stouw

**Affiliations:** 1 Saffier Location Domus Nostra, Korsakoff Expertise Center, 2555 XZ The Hague, The Netherlands; 2 Music Therapy Department, ArtEZ University of the Arts, 7511 PN Enschede, The Netherlands; g.vanderstouw@artez.nl; 3 ZorgAccent Location Krönnenzommer, Korsakoff Expertise Center, 7447 PK Hellendoorn, The Netherlands

**Keywords:** music therapy, interventions, Korsakoff’s syndrome, alcohol abuse, memory disorder, empathic directive approach, quality of life

## Abstract

In this perspective article, the authors give insight into the beneficial effects and the current developments in music therapy for patients with Korsakoff’s syndrome (KS) in the Netherlands. Music may be the key to distracting patients from negative moods, to help them express emotions and to teach them new skills on physical, psychosocial and cognitive levels. This may lead to improving the quality of life of patients with KS. Emphasis is placed on the personal experience of the authors and on the future directions in the field. Their experience, as well as the experience of music therapy colleagues working in the field with the same target population (joined together in the Music Therapy Korsakoff Expertise Group), is situated in the context of existing literature and showcases current developments in the specific field of music therapy and KS. Since literature on this specific topic is limited, the authors allowed themselves to delve into somewhat older but still leading and representative literature. There is still little knowledge on how music therapy may contribute to reducing the impairments patients with KS suffer and to improving their quality of life in general. Using the Empathic Directive Approach (EDA) as the starting point, the authors elaborate on different potential approaches and interventions. With this article, the authors aim to gain more insight into the potential role of the music therapist by highlighting music–therapeutic micro-interventions and to provide recommendations for future directions on how to integrate music therapy in the treatment of patients with Korsakoff’s syndrome.

## 1. Introduction

In September 2022, a group of 15–20 music therapy clinicians in the Netherlands who all work with patients with Korsakoff’s syndrome joined together and established the “Music Therapy Korsakoff Expertise Group”. To date, this network has organized two physical meetings where participants shared experiences and elaborated on the different goals and interventions for the treatment of the target group. In addition, the group discussed the future prospectives of their profession. In between the physical meetings, the online discussion continued, being an ongoing process. This article is (partly) based on these discussions and reflects the opinion of the by now 30 members of this expertise group.

## 2. Korsakoff’s Syndrome

The origin of the name “*Korsakoff’s syndrome*” (KS) stems from the Russian neuropsychiatrist Sergei Korsakoff, who first described a patient in 1887 as somebody with “*a form of psychic disorder which occurs in conjunction with multiple neuritis*”. KS is a disorder that primarily affects the memory system in the brain. It usually results from a deficiency of thiamine (vitamin B1) mainly caused by alcohol abuse, but also due to dietary deficiencies, extreme and prolonged vomiting, eating disorders or as an effect of chemotherapy [1,2,3].

“*…Drinking alcohol excessively over a prolonged period of time may lead to (severe) brain damage. This can be caused by the toxic effects of alcohol on nerve cells, head injury and blood vessel damage.*” [4] (p. 115). In addition to KS, there is another type of alcohol-related brain damage called Wernicke’s encephalopathy (WE). If acute WE is not treated, 20% of the cases proceed to death, and 85% of the “survivors” develop KS. Up to 25% of these survivors do not show any improvement in cognitive functioning and will require long-term care [4,5,6,7,8].

One of the most prominent symptoms of KS is gaps in memory, which are usually filled inaccurately (confabulation), and difficulty in learning new information. Besides these severe memory disorders and confabulations, patients with KS have difficulties with central executive functions and have little awareness of their capabilities and limitations [9]. These problems manifest themselves in having difficulties with planning and organizing activities in daily life. Therefore, patients with KS have a strong need for structure in their daily life. Based on the problems patients with KS face on a daily basis, many situations can trigger stress. This stress can negatively influence the mood of patients with KS. In the long term, this can negatively influence their quality of life [6,10,11].

The literature about KS in terms of prevalence and incidence is limited. In the Netherlands, the home country of the authors, the prevalence is estimated to be 48 per 100,000 inhabitants. This brings the total estimated number of patients suffering from KS in NL to 8000 to 10,000, given the fact that NL has 17.5 million inhabitants [4,11]. Since many alcohol-addicted people are healthcare-avoiders, this number may not be accurate.

Patients with KS often need 24 h supervision, care, and guidance. In the Netherlands, there are over 40 organizations affiliated with the national Korsakoff Centre of Excellence; about 15 of them have accreditation as a Korsakoff Expertise Centre. The mean age of admission in a long term care facility (LTCF) is 56.7 years, and the mean length of stay is 6 years [4,6,10,11].

In conclusion: the daily life for patients with KS can be hard due to the problems they face when it comes to taking initiative, solving problems and memory tasks. Patients with KS often lack insight into their own problems, which is the reason why many of them feel that they live unjustly in an LTCF. This can have a major impact on their mood and quality of life, and one of the reasons for the low scores of the quality of life that were found [12]. As a result of the impairments patients with KS face on a daily basis, caring for them is challenging. Therefore, over the past decades, specific approaches have been developed to give patients with KS the most suitable care and treatment.

## 3. The Empathic Directive Approach (EDA)

The most prominent approach, specifically developed for patients with KS, is the Empathic Directive Approach (EDA). The EDA, developed in the Netherlands, is an approach based on a positive and supportive attitude, commonly used in the treatment of people with KS. It is built on the 5-C/K model, which comprises five directives: creative, concrete, consistent, continuous and short (the Dutch word for short is “kort”) [13,14]. It has similarities with behavioural-based approaches. By offering instructions in exactly the same way each time, conditioning principles and forms of implicit learning are being used. By so doing, the therapist reinforces the desired behaviour and ignores unwanted behaviour. The relevance of this best practice is recognized by the Dutch Centre of Knowledge for Korsakoff’s syndrome (KKC) [15], which states that positive reinforcement is more effective than correcting, confronting, or an overly direct approach.

## 4. Music Therapy (MT)

“*In the attempt to meet the needs of the individual, the challenge is to find adequate responses and to develop relevant and effective therapeutic strategies*” [16]. One of the non-pharmaceutical treatments offered to patient with KS in an LTFC is music therapy.

Music may be the key to distracting patients from negative moods, to helping them express emotions, and to teaching them new skills on physical, psychosocial and cognitive levels. This may lead to improving the quality of life of patients with KS.

The American Music Therapy Association defines music therapy as “*…the clinical and evidence-based use of music interventions to accomplish individualized goals within a therapeutic relationship by a credentialed professional who has completed an approved music therapy program…*” [17]. These goals strive to obtain change, development, stabilization, or acceptance in the emotional, behavioural, cognitive, social, neurological or physical realms [18].

A music therapist offers an indicated treatment, taking into account the (developmental) possibilities, requests for help and preferences of the patient. In the treatment, the music therapist uses music therapeutic methodologies, methods and techniques, such as (together) improvising, receptive music listening, composing, songwriting, playing, singing or moving to music and reflecting on musical actions in relation to the request for help. Working from the capacities of a patient, the therapist helps to strengthen his possibilities by acting musically within the therapeutic context and relationship. In doing so, the music therapist stimulates the transfer of the possibilities developed within music therapy to other areas of the patient’s life [17,19].

## 5. Literature Review on Music Therapy and Korsakoff’s Syndrome

Music therapy may be an effective treatment that can reach patients with KS [2,5]. Literature about this specific subject is scarce. To ensure no article or book chapter about this topic was missed, a systematic literature search was conducted for any related publication, published up to 2022, in the following databases: PubMed, APA PsycINFO/PsycIndex, EBook Central, eBook Academic Collection, EBSCO Host Platform, Google Scholar. The following (Dutch and English) terms were included in the search: music, music therapy, arts therapy, Korsakoff, Wernicke, alcohol dementia. The references used in this article were purposefully limited to publications that focused specifically on these terms.

Furthermore, (hand)searches were carried out in specific music(therapy) databases, such as the *Nordic Journal of Music Therapy* and *Journal of Music Therapy*. Reference lists of the relevant publications were reviewed. Finally, conference proceedings and (unpublished) dissertation/abstracts were reviewed.

Titles and abstracts of the references retrieved from the searches were screened by two reviewers independently (HvW and MvBR), according to the selection criteria. Full texts were obtained if the articles met the aforementioned criteria.

Each relevant article was put under the magnifying glass in that we specifically looked for leads how the EDA is applied in the music therapy interventions.

## 6. Results

Van Bruggen-Rufi [17] and Van Straaten [20] have written guidelines for music therapy with KS patients. The first guideline focusses on reducing problem behaviour, the second one on stimulating and improving executive functions. Van Bruggen-Rufi [17] specifically mentions that the EDA can be used in various interventions, like listening to preferred music, singing along or playing with an existing song or free improvisation, using specific music therapy techniques [21,22].

In her bachelor’s thesis, Dinghs developed a music therapy method based on the aforementioned guidelines and on the Empathic Directive Approach for patients with KS [23]. The interventions have been worked out to inspire other music therapists to apply them, with the intention being to gain more self-esteem and reinforce social interaction. The effects of this method have not been studied or implemented yet.

Navone [24] conducted a single case study to explore the effect of a music therapy intervention in a patient with KS who suffers from depression. He concluded that “*…music therapy treatment shows its effects on areas involved in emotional processing and regulation…music therapy can therefore be an effective intervention for improving the quality of life and supporting caregivers in the management of patients with Korsakoff’s syndrome*” [24]. Navone does not mention the EDA specifically.

The first music therapy micro-intervention in the Netherlands was published in 2020 by Van der Eng and Hakvoort, aiming to stimulate the executive functions in patients with Korsakoff’s syndrome [25]. The authors report on a music therapy micro-intervention for patients with KS who showed behavioural problems due to apathy. The intervention aims to improve the patient’s attention and activation through playing familiar songs, leading to free improvisation. By so doing, the patient is stimulated to take initiative. The working mechanisms of music in the brain are explained.

Van der Stouw-Dannenberg conducted an exploratory case study in 2021 on the effects of music therapy on stress, mood and quality of life in patients with KS [26]. A quantitative single group design was conducted. Participants (*n* = 2) received bi-weekly, individual active music therapy sessions over a period of 6 weeks. Effects on stress, mood and quality of life were measured using the VAS, Interact and Qualiko. The VAS showed significant stress reduction and mood improvement after therapy sessions. No significant effects were found on the Interact and Qualiko on stress reduction, mood and quality of life.

In 2021, a case report article was published by Van Bruggen-Rufi and Van Rijn [5]. The authors present two different cases in which it is clear how music therapy was beneficial for two patients with KS e struggling with behavioural and emotional problems.

Another case report article was published in 2022 by Van Bruggen, Benkmil et al. [2]. This article does not only report on music therapy, but on creative arts therapy and dance/movement therapy in general. Five case vignettes give the reader insight into the beneficial effects of creative arts therapy. By so doing, the authors hope to break the stigma that haunts almost all KS patients: “*the low-life-drunks who made a big mess of their life*”.

Rita Kárpáti carried out a brief survey in 2023among music therapists in the Netherlands, specialized in Korsakoff care (*n* = 13), focusing on their attitudes towards patient autonomy in the long-term care facility environment [27]. The results of this survey were presented at the 2023 Online Conference for Music Therapy. Despite the great diversity in the respondents’ methodological preferences, all respondents familiar with the Empathic Directive Approach praised the Dutch method’s practical usefulness and emphasized the importance of acting consistently with the established house rules and multidisciplinary agreements. At the same time, their answers hinted at a unique niche for music therapy within the healthcare team when it comes to offering personalized care, a concept in line with the current shift towards client-cantered care in the Dutch medical–legal discourse [28].

By focusing on the patient’s personal preferences and choices, attuning to the patient’s needs on a momentary basis and utilizing the flexibility of the therapeutic environment, the music therapists supported their patients in their sense of control and agency challenged by the myriad of semi-voluntary medical procedures they had been facing since their admission. Interventions using personalized playlists, group improvisations involving conducting and changing leadership roles and adaptive songwriting and recording techniques were mentioned as practical ways to achieve this, while community-building performance projects were used to break the imaginary “prison walls” between the resident, nursing home and the outside world [27].

Finally, in 2023, Van der Stouw and Hakvoort described another micro-intervention for patients with KS. This micro-intervention is based on the EDA and forms of implicit learning. The micro-intervention describes step-by-step how to learn a basic drum rhythm and uses this rhythm in an improvisation for stress reduction [29].

In conclusion, it is safe to say that although the literature on music therapy and Korsakoff’s syndrome is limited, the results of the literature search show that music therapy is a fast-growing treatment that has already been established in the Netherlands but not so much on an international level yet. Most articles are case reports or focus on small, mostly qualitative studies. The expertise of Dutch music therapy colleagues working with the same target population (joined together in the Music Therapy Korsakoff Expertise Group) indicates that when treating patients with KS, finding the most suitable intervention to meet their needs is challenging.

Taking the EDA in mind, this approach might very well be combined with already existing behaviourally focused music therapy interventions for patients with KS. The joint music therapy expertise group has embraced the EDA and believes that combining these methods within micro-interventions is very promising. Their future meetings will therefore be used to develop and describe commonly used micro-interventions. But what does the micro-intervention exactly entail?

## 7. Micro-Intervention

A music therapy micro-intervention can be considered as a short part of a session in which the music therapist uses specific therapeutic techniques or steps to work on specific patient goals [25,30,31]. Micro-interventions are systematically described and follow a step-by-step approach based on both recent theoretical models as well as the latest scientific evidence. The development of music therapy micro-interventions is important to music therapy practice because, on the one hand, the way of intervening in micro-interventions is strongly linked to core components of music therapy and, on the other hand, because describing interventions helps to further develop the profession [30,31].

Micro-interventions focus on the detailed role of music within the treatment and the core neurological mechanisms that need to be triggered to provide maximal results on a micro-level. It offers a detailed description on how to apply music scientifically and neurologically to optimize its impact during a specific music therapeutic intervention. By so doing, the intervention can be applied in all settings all over the world. Hence, describing micro-interventions may also stimulate the transferability of valuable clinical practices which in turn may strengthen thinking about the relationship between clinical practice, theory and research [30]:

“*The format of this type of intervention allows music therapists to describe music therapy interventions in detail, including the intervention and its musical working-mechanisms. The format is intended to guide music therapists to execute their intervention in comparable manners, which in the case of a scientific study could improve research fidelity. Development and dissemination of music therapy micro-interventions could lead to improved research outcomes and strengthen the evidence-based foundation of the profession.*”

[31] (p. 86).

## 8. Integrating EDA and Music Therapy

The joint Music Therapy Korsakoff Expertise Group has embraced the EDA within their work. During their meetings, they exchanged experiences and discussed how the EDA is applied in commonly used interventions by patients with KS. The majority of the members indicated that they all use the EDA in their work, but not all of them are aware of this. Recently, a small study has taken place in which five music therapists in the field went through an in-depth interview about how they integrate the EDA within their daily work. Useful insight in the clinical work of those interviewed, and the most commonly used techniques and methods they use on a day-to-day basis, was gained. This information may be the starting point of developing new micro-interventions, which will be the top priority of the MT Expertise Group.

In 2013, Dinghs [23] wrote:

*“…Clients with KS often have to cope with feelings of insecurity, performance anxiety or guilt. These feelings are due to memory disorders, central executive disorders, psychiatric and physical disorders and often lead to problem behavior. The Empathic Directive Approach is effective for building a relationship of trust between the caregiver and the client and can also help to bring structure in the client’s daily life. Music has the power to (non-verbally) express and provoke emotions. Music therapists can build a relationship of trust with the client by using specific musical parameters. From this point, the therapist can give structure in the music itself and through the course of the music therapy. By using musical therapeutic activities, where the client experiences success, the client’s self-esteem and social interaction can be improved.”*

[23] (p. 7).

Dinghs was the first to describe how to implement the EDA into the daily music therapy practice by means of four of Bruscia’s clinical techniques for improvisational music therapy: these are the techniques of empathy, of structuring, of elicitation and of redirection [21,22]. Her recommendation to execute quantitative research in the future, studying the effects of a music therapy intervention using the EDA, has not been acted upon yet, which is a missed opportunity.

In music therapy, the music can be used both empathetically and as a directive to encourage a patient to take a certain action. Several studies show that music generally modulates activity in brain structures such as the amygdala and the mesolimbic reward system. These systems are involved in emotional and motivational processes [32,33,34,35]. For example, being passive (listening) or actively engaged in music (creating) increases blood flow to the pleasure centres of the brain, increasing endorphin production and decreasing cortisol production, helping patients to activate and put the patient in a state where they are ready to act [36,37].

As for the directive part of the EDA, music has important qualities to structure, especially when it is goal-oriented used by a music therapist. For example, the use of rhythmic entrainment means that physical rhythms such as heart rate or breathing adapt to the tempo at which the patient plays a rhythm [38]. This principle helps the patient to get into a musical flow. The music therapist can use this entrainment to direct the patient to a certain level of activity or relaxation [29].

In short: the use of the EDA in music therapy for patients with KS is already a relatively common approach [23]. During the meeting of the Korsakoff Expertise Group, most of the music therapists were already familiar with the EDA and applied this in their practice. The following step is to investigate what the similarities are when it comes to working with EDA in music therapy and what is the effect of this approach.

## 9. Conclusions and Recommendations

In recent years, the description micro-interventions is up and coming within the clinical practice of music therapists. Developing consensus-based music therapeutic micro-interventions, based on the Empathic Directive Approach, may be the first step toward more quantitative research. During the second network meeting of the Music Therapy Korsakoff Expertise Group in February 2023, the importance of (developing) micro-interventions formed the main topic of the meeting.

The group will continue collaborating and exchanging experience and knowledge. By developing micro-interventions together, a consensus-based practice will be created. Once a few micro-interventions have been developed, they can be used as a blueprint for quantitative research. It is recommended to continue developing micro-interventions and to collect them, so that all music therapists can apply them into their clinical work in exactly the same way.

The expertise group will elaborate on this in the near future and specify how the EDA, combined with other music therapy methods, is integrated in their daily work.

## 10. Future Perspectives: From Practice-Based Evidence towards Evidence-Based Practice

Once a collection of different micro-interventions has been accumulated, these micro-interventions can be implemented and studied in the clinical practice. Plans have been made to design a multi-centre research work studying the effects of music therapy for people with KS through a combination of the Empathic Directive Approach and such consensus-based micro-interventions. The results gained from this study will help us to tailor-make music therapy practices to the specific needs of patients with KS.

The results of the study will give more insight into the effects of music therapy on different treatment goals within the target population of patients with Korsakoff’s syndrome, providing a clear direction for the further development of the profession. By doing so, music therapy will contribute to changing practice-based evidence into evidence-based practice.

## Data Availability

Not applicable.
